# Multidrug-resistant and extensively drug-resistant *Neisseria gonorrhoeae* in Canada, 2012–2016

**DOI:** 10.14745/ccdr.v45i23a01

**Published:** 2019-02-07

**Authors:** I Martin, P Sawatzky, V Allen, B Lefebvre, LMN Hoang, P Naidu, J Minion, P Van Caeseele, D Haldane, RR Gad, G Zahariadis, A Corriveau, G German, K Tomas, MR Mulvey

**Affiliations:** 1 Bacterial Pathogens Division, National Microbiology Laboratory, Public Health Agency of Canada, Winnipeg, MB; 2 Public Health Ontario Laboratories, Toronto, ON; 3 Laboratoire de santé publique du Québec, Ste-Anne-de-Bellevue, QC; 4 British Columbia Centre for Disease Control Public Health Laboratory, Vancouver, BC; 5 Provincial Laboratory for Public Health, Edmonton, AB; 6 Roy Romanow Provincial Laboratory, Regina, SK; 7 Cadham Provincial Laboratory, Winnipeg, MB; 8 Queen Elizabeth II Health Sciences Centre, Halifax, NS; 9 New Brunswick Department of Health, Fredericton, NB; 10 Newfoundland and Labrador Public Health Laboratory, St. John’s, NL; 11 Department of Health and Social Services, Government of the Northwest Territories, Yellowknife, NT; 12 Health PEI, Charlottetown, PE; 13 Centre for Communicable Diseases and Infection Control, Public Health Agency of Canada, Ottawa, ON

**Keywords:** *Neisseria gonorrhoeae*, antimicrobial resistance, laboratory surveillance, *N. gonorrhoeae* multidrug resistant

## Abstract

**Background:**

*Neisseria gonorrhoeae* have acquired resistance to many antimicrobials, including third generation cephalosporins and azithromycin, which are the current gonococcal combination therapy recommended by the *Canadian Guidelines on Sexually Transmitted Infections*.

**Objective:**

To describe antimicrobial susceptibilities for *N. gonorrhoeae* circulating in Canada between 2012 and 2016.

**Methods:**

Antimicrobial resistance profiles were determined using agar dilution of *N. gonorrhoeae* isolated in Canada 2012–2016 (n=10,167) following Clinical Laboratory Standards Institute guidelines. Data were analyzed by applying multidrug-resistant *gonococci* (MDR-GC) and extensively drug-resistant *gonococci* (XDR-GC) definitions.

**Results:**

Between 2012 and 2016, the proportion of MDR-GC increased from 6.2% to 8.9% and a total of 19 cases of XDR-GC were identified in Canada (0.1%, 19/18,768). The proportion of isolates with decreased susceptibility to cephalosporins declined between 2012 and 2016 from 5.9% to 2.0% while azithromycin resistance increased from 0.8% to 7.2% in the same period.

**Conclusion:**

While XDR-GC are currently rare in Canada, MDR-GC have increased over the last five years. Azithromycin resistance in *N. gonorrhoeae* is established and spreading in Canada, exceeding the 5% level at which the World Health Organization states an antimicrobial should be reviewed as an appropriate treatment. Continued surveillance of antimicrobial susceptibilities of *N. gonorrhoeae* is necessary to inform treatment guidelines and mitigate the impact of resistant gonorrhea.

## Introduction

Gonorrhea is the second most commonly reported sexually transmitted infection in Canada, the causative organism being *Neisseria gonorrhoeae*. In 2016, 23,708 cases of gonorrhea were reported to the Public Health Agency of Canada (PHAC); rates had increased 87%, from 34.9 cases/100,000 population in 2012 to 65.4 cases/100,000 population in 2016 (1). In 2016, 82% of the total reported cases of gonorrhea in Canada occurred in the 15–39 year age group and the highest rates among males were found among those aged 20–29 years and among females aged 15–24 years (2). Globally, there are an estimated 78 million cases of gonorrhea infection occurring per year (3). Treatment is complicated, as *N. gonorrhoeae* have acquired resistance mechanisms to many of the antimicrobials used for treatment over the years (4). This resistance has been documented by surveillance programs that are used to support appropriate treatment recommendations.

A challenge to gonococcal (GC) surveillance programs is that the number of cultures available for antimicrobial susceptibility testing is on the decline due to the shift from the use of bacterial culture to nucleic acid amplification test (NAAT) for the diagnosis of gonorrhea. This is of concern as *N. gonorrhoeae* cultures are also required for antimicrobial susceptibility testing. Currently almost 80% of gonococcal infections in Canada are now diagnosed using NAAT (5). Some jurisdictions in Canada no longer maintain the capacity to culture this organism and, therefore, antimicrobial susceptibility data in these jurisdictions are not available.

Canadian gonococcal surveillance data from 2012 reported an increase in isolates with decreased susceptibility to cephalosporins, prompting an update to the recommendation for gonorrhea treatment in the *Canadian Guidelines on Sexually Transmitted Infections* to combination therapy with two antibiotics. In uncomplicated anogenital infections and pharyngeal infections, ceftriaxone 250 mg intramuscularly (IM) plus azithromycin 1 g orally is currently recommended as a first-line treatment (6).

Along with rising antimicrobial resistance rates, there have also been reports of *N. gonorrhoeae* with high-level resistance and gonococcal treatment failures; all causes for concern. Treatment failures involving cefixime, a potent oral cephalosporin, have been reported internationally (7–12) as well as in Canada (13,14). Most of these cases were successfully treated with ceftriaxone (250 mg IM). In 2009, Japan identified an isolate (H041) that caused a pharyngeal treatment failure with ceftriaxone that showed unusually high minimum inhibitory concentrations (MICs) to ceftriaxone (2 mg/L) and cefixime (8 mg/L); treatment with ceftriaxone 1 g intravenously cleared the infection (15). More pharyngeal treatment failures to ceftriaxone were reported in Sweden (16,17), Slovenia (18) and Australia (19,20), which were then treated successfully with a higher dosage of ceftriaxone (1 g IM), azithromycin (2 g orally) or a combination of ceftriaxone (250 mg IM) and azithromycin (1 g orally). In 2011, France reported the first genital treatment failure to ceftriaxone in Europe (11). In 2014, the first dual antimicrobial therapy treatment failure was reported in the United Kingdom (UK) (ceftriaxone 500 mg plus azithromycin 1 g) and was successfully treated with ceftriaxone (1 g IM) plus azithromycin (2 g oral) (21). Since 2013, cases of ceftriaxone resistance have been identified and characterized in a number of countries, including Canada, Japan and Australia, which were successfully treated with azithromycin (22,23). The UK and Australia have also recently reported treatment failure cases due to high-level ceftriaxone resistance (MIC=0.5 mg/L) and high-level azithromycin resistance (MIC greater than or equal to 256 mg/L). The UK case was successfully treated with intravenous ertapenem (24).

Rising azithromycin resistance rates have also been reported in Canada (5) and internationally (25), which is of concern as azithromycin is part of the recommended combination therapy. Along with increasing moderate-level azithromycin resistance, there have been reports of high-level azithromycin resistance (MIC greater than or equal to 256 mg/L) that were associated with a large-scale outbreak in the UK (26). Although isolates with this high azithromycin MIC have been identified in Canada, a total of seven were identified between 2009 and 2016 (5); these cases appear to be sporadic occurrences in Canada and have not spread.

In 2009 (27), definitions were established for multidrug-resistant gonococci (MDR-GC) and extensively drug-resistant gonococci (XDR-GC), which we have recently updated, taking into account the *Canadian Guidelines on Sexually Transmitted Infections* and the antimicrobials being tested in our routine laboratory surveillance (**Text box 1**).

**Text box 1: Definitions of multidrug-resistant gonococci (MDR-GC) and extensively drug-resistant gonococci (XDR-GC)**

**MDR-GC** – decreased susceptibility/resistance to ***one*** currently recommended therapy (cephalosporin **OR** azithromycin) PLUS resistance to at least ***two*** other antimicrobials (penicillin, tetracycline, erythromycin, ciprofloxacin)

**XDR-GC** – decreased susceptibility/resistance to ***two*** currently recommended therapies (cephalosporin **AND** azithromycin) PLUS resistance to at least ***two*** other antimicrobials (penicillin, tetracycline, erythromycin, ciprofloxacin)

PHAC’s National Microbiology Laboratory (NML), in collaboration with the provincial laboratories, has been monitoring the antimicrobial susceptibilities of *N. gonorrhoeae* since 1985. In this report, we present national-level trends in antimicrobial susceptibilities of *N. gonorrhoeae* collected from 2012 to 2016, applying the updated MDR-GC and XDR-GC definitions.

## Methods

Between 2012 and 2016, *N. gonorrhoeae* cultures were submitted to the NML by provincial laboratories when they identified a resistant (R) isolate or by laboratories that did not conduct antimicrobial susceptibility testing (**Table 1**). Information regarding the isolates submitted to NML included sex and age of the patient, province/territory where infection was diagnosed, as well as the site of infection. Annually, each province/territory informs the NML of the total number of cultures collected and tested, either in their province/territory or at the NML (Table 1). These totals are used as the denominators in determining the proportions of antimicrobial drug resistance.

**Table 1:** Neisseria gonorrhoeae cultures collected by provinces and territories and sent to the National Microbiology Laboratory (NML), 2012–2016

Year	Cultured	BC^a^	AB^a^	SK^b^	MB^b^	ON^a^	QC^a^	NS^b^	Other^b,c^	Total cultures	Total cases reported in Canada	% of total cases tested by cultures	
**2012**	**Collected**	372	497	57	49	1,218	838	0	5	**3,036**	**12,561**	**24.20%**
	**Sent to NML^d^**	92	93	57	8	396	383	0	4	**1,033**		
**2013**	**Collected**	454	514	69	29	1,404	716	1	8	**3,195**	**13,786**	**23.20%**
	**Sent to NML^d^**	170	135	67	7	498	298	1	8	**1,184**		
**2014**	**Collected**	492	468	91	46	1,767	918	15	12	**3,809**	**16,285**	**23.40%**
	**Sent to NML^d^**	335	323	91	46	849	400	14	12	**2,070**		
**2015**	**Collected**	602	793	62	48	1,673	986	13	13	**4,190**	**19,845**	**21.10%**
	**Sent to NML^d^**	387	512	65	44	1,076	531	13	10	**2,638**		
**2016**	**Collected**	600	786	86	85	1,735	1,197	32	17	**4,538**	**23,708**	**19.10%**
	**Sent to NML^d^**	348	695	85	81	1,068	927	31	7	**3,242**		
**Total**	**Collected**	2,520	3,058	365	257	7,797	4,655	61	55	**18,768**	**86,185**	**21.80%**
	**Sent to NML^d^**	1,332	1,758	365	186	3,887	2,539	59	41	**10,167**		

Abbreviations: AB, Alberta; BC, British Columbia; MB, Manitoba; NS, Nova Scotia; ON, Ontario; QC, Quebec; SK, Saskatchewan

^a^ Province performs antimicrobial susceptibility testing and sends only primarily resistant isolates to the NML

^b^ Province does not perform antimicrobial susceptibility testing (Manitoba stopped in 2014) and sends all cultures to the NML

^c^ Other includes Northwest Territories, New Brunswick, Newfoundland and Prince Edward Island. Nunavut and the Yukon did not report or send any *N. gonorrhoeae* cultures to the NML from 2012 to 2016

^d^ Numbers include only one culture/case

Antimicrobial susceptibilities of *N. gonorrhoeae* to azithromycin, cefixime, ceftriaxone, erythromycin, penicillin, spectinomycin, tetracycline, ciprofloxacin, ertapenem and gentamicin were determined using agar dilution (28). The MIC interpretative standards used were as recommended by the Clinical and Laboratory Standards Institute (28) except for erythromycin (R ≥ 2 mg/L) (29), azithromycin (R ≥ 2 mg/L) (30), ceftriaxone (DS ≥ 0.125 mg/L) and cefixime (DS ≥ 0.25 mg/L) (31), ertapenem (NS ≥ 0.063 mg/L) (32) and gentamicin (R ≥ 32 mg/L) (33,34). The *N. gonorrhoeae* reference cultures ATCC49226, WHOF, WHOG, WHOK, and WHOP/WHOU were used as controls. Statistical analysis was determined by using EpiCalc 2000 version 1.02 (www.brixtonhealth.com/epicalc.html).

A 2 × 2 χ^2^ test was used to compare proportions of resistance per year to identify significant differences between years (*p* values calculated with 99% confidence intervals).

## Results

From 2012 through 2016, 21.8% (n=18,768) of the 86,185 cases of *N. gonorrhoeae* infection reported in Canada (1) were diagnosed by culture. Provincial public health laboratories submitted 10,167 isolates to NML for testing (2012, n=1,033; 2013, n=1,184; 2014, n=2,070; 2015, n=2,638; 2016, n=3,242). Sex and age data of patients were available for 10,104 (99.4%) isolates. Of these, 8,649 (85.6%) were from male patients (median age 30 years; range less than 1–83 years) and 1,455 (14.4%) were from female patients (median age 26 years; range less than 1–71 years). Source specimens included urethral (n=4,836), rectal (n=2,100), pharyngeal (n=1,367), cervical (n=625), vaginal (n=249) and other sources (n=209); sources for 781 isolates were not given. The sexual orientation of patients and information on cases of treatment failure were not available.

### Multidrug-resistant gonorrhea

**Figure 1:**
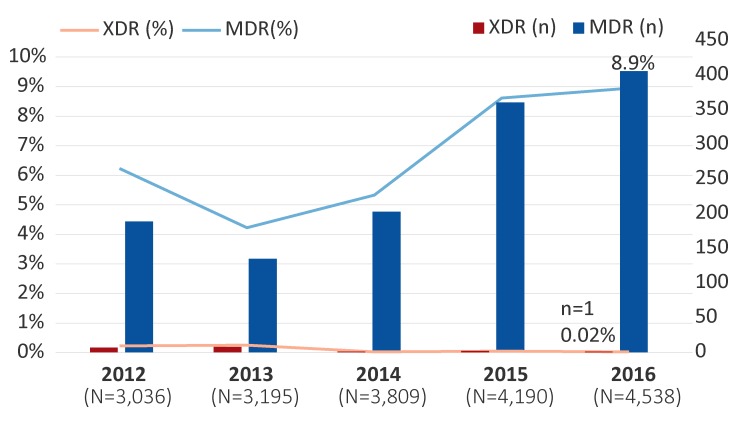
Multidrug-resistant and extensively drug-resistant Neisseria gonorrhoeae isolates in Canada, 2012–2016^a^ Abbreviations: MDR, multidrug-resistant gonococci; n, number; N, total number; XDR, extensively drug-resistant gonococci ^a^ Percentages are based on the total number of isolates tested nationally per year

The proportion of MDR-GC increased from 6.2% (n=189/3,036) in 2012 to 8.9% (n=406/4,538) (*p*<0.001) in 2016. These percentages represent the proportion of isolates with decreased susceptibility to the cephalosporins or resistance to azithromycin, along with resistance to two other antimicrobials (**Figure 1**).

**Figure 2:**
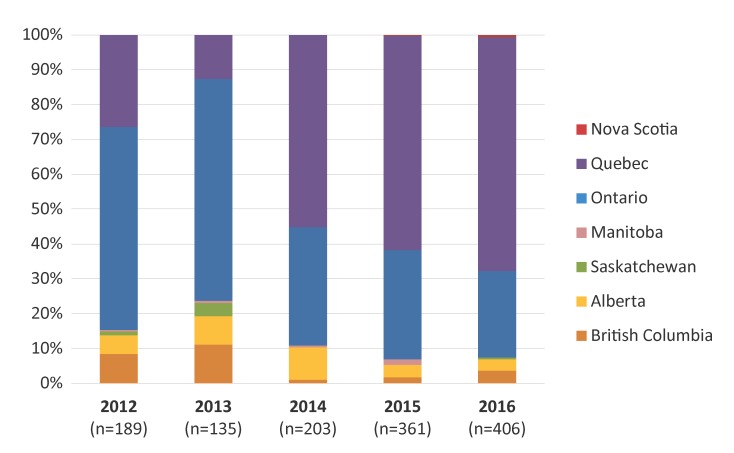
Provincial distribution of multidrug-resistant gonococci by year, 2012–2016^a^ Abbreviation: n, number ^a^ Percentages are based on the total number of multidrug-resistant gonococci identified each year

Provincial distribution of MDR-GC identified in Canada is represented in **Figure 2**, with the highest proportion identified in Quebec (67.0%), followed by Ontario (24.9%) in 2016. British Columbia, Alberta, Nova Scotia and Saskatchewan also identified cases of MDR-GC in 2016.

**Figure 3:**
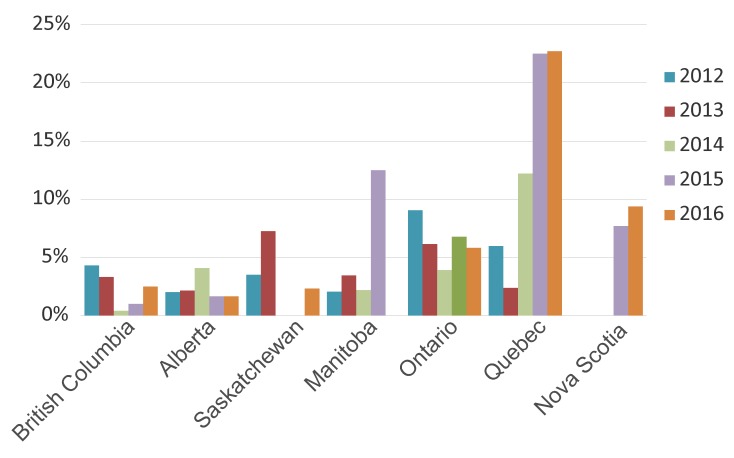
Proportion of multidrug-resistant gonococci in each province from 2012 to 2016^a^ ^a^ Percentages are based on the total number of cultures in each province

The temporal trends of MDR-GC within each province are displayed in **Figure 3**, and the provinces with the highest proportions of MDR-GC in 2016 were Quebec (22.7%) followed by Nova Scotia (9.4%) and Ontario (5.8%).

**Figure 4:**
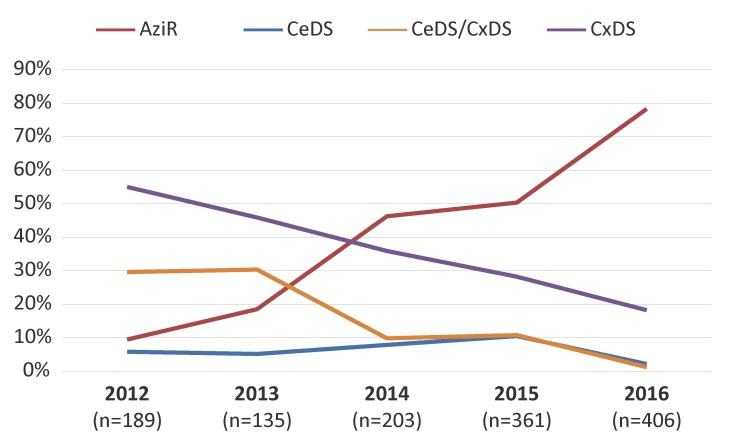
Trends of antimicrobials associated with multidrug-resistant gonococci, 2012–2016^a^ Abbreviations: AziR, azithromycin resistant; CeDS, decreased susceptibility to cefixime; CeDS/CxDS, decreased susceptibility to cefixime and ceftriaxone; CxDS, decreased susceptibility to ceftriaxone; n, number ^a^ Percentages based on total number of multidrug-resistant gonococci per year

**Figure 4** represents the trends of the antimicrobials associated with MDR-GC. The MDR-GC associated with azithromycin resistance increased significantly (*p*<0.001) from 9.5% in 2012 to 78.3% in 2016. Conversely, MDR-GC associated with decreased susceptibility to cefixime and ceftriaxone declined significantly (*p*<0.001) from 29.6% in 2012 to 1.2% in 2016.

**Figure 5:**
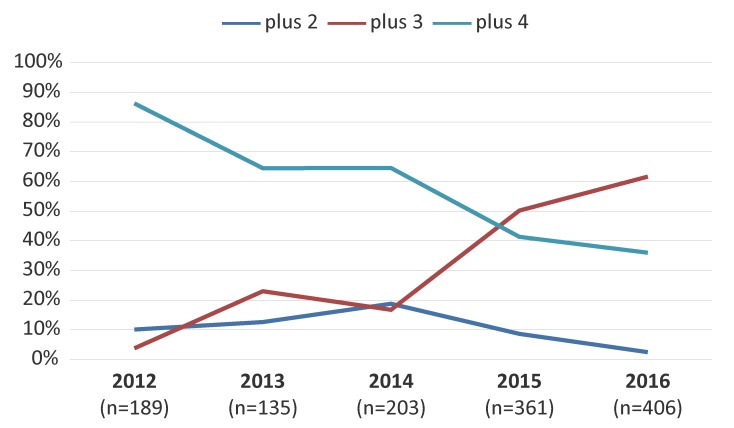
Trends of multidrug-resistant gonococci with resistance to two, three or four additional antimicrobials^a^ Abbreviations: n, number; plus 2, multidrug-resistant gonococci with resistance to two antimicrobials not recommended for therapy; plus 3, multidrug-resistant gonococci with resistance to three antimicrobials not recommended for therapy; plus 4, multidrug-resistant gonococci with resistance to four antimicrobials not recommended for therapy ^a^ Percentages based on total number of multidrug-resistant gonococci per year

**Figure 5** represents the trends of MDR-GC associated with resistance to two, three or four additional antimicrobials. The MDR-GC with resistance to three additional antimicrobials increased significantly (*p*<0.001) from 3.7% in 2012 to 61.6% in 2016 with ciprofloxacin, erythromycin and tetracycline as the most common co-resistance antimicrobials.

### Extensively drug-resistant gonococci

From 2012 to 2016, only 19 cases of XDR-GC were identified in Canada (0.1%, n=19/18,768). In 2012, seven XDR-GC isolates with combined decreased susceptibility to cephalosporins and resistance to azithromycin were identified (0.2%, n=7/3,036; Ontario n=6; British Columbia n=1), which increased to eight (0.3%, n=8/3,195; Ontario n=5; British Columbia n=2; Saskatchewan n=1) in 2013. From 2014 to 2016, however, XDR-GC numbers were lower: in 2014, only one was identified (0.03%, n=1/3,809; Quebec); in 2015, two were detected (0.05%, n=2/4,190; Ontario n=1; Quebec n=1); and in 2016, only one XDR-GC was isolated (0.02%, n=1/4,538; British Columbia) (Figure 1).

### Trends in resistance patterns

The proportion of *N. gonorrhoeae* that were identified as susceptible to all antimicrobials tested declined significantly (*p*<0.001) from 67.5% in 2012 to 35.4% in 2016.

**Figure 6:**
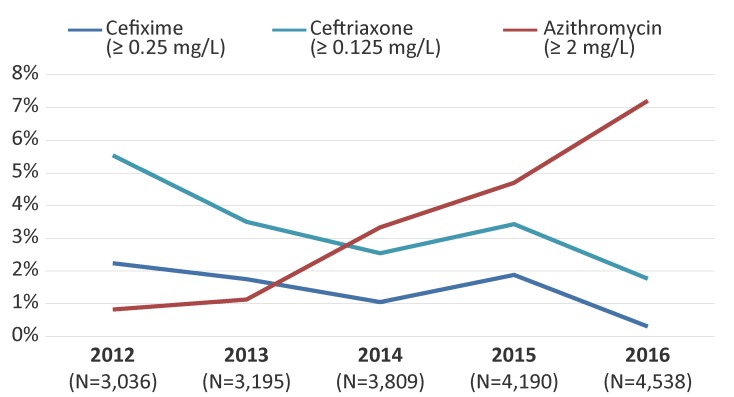
Decreased susceptibility to cefixime and ceftriaxone and resistance to azithromycin for Neisseria gonorrhoeae isolates in Canada, 2012–2016^a^ Abbreviations: mg/L, milligrams per litre; N, total number; ≥, superior or equal to ^a^ Percentage based on total number of isolates tested nationally

In 2012, 2.2% (n=68/3,036) of isolates had decreased susceptibility to cefixime. This proportion has decreased significantly (*p*<0.001) to 0.3% (n=14/4,538) in 2016 (**Figure 6**). Similarly, decreased ceftriaxone susceptibility was 5.5% (n=168/3,036) in 2012 and decreased significantly (*p*<0.001) to 1.8% (n=80/4,538) by 2016 (Figure 6).

The proportion of azithromycin resistance increased significantly (*p*<0.001) from 0.8% (n=25/3,036) in 2012 to 7.2% (n=327/4,538) in 2016 (Figure 6). The modal MICs of isolates resistant to azithromycin decreased from 8 mg/L between 2012 and 2014 to 2 mg/L in 2015 and 2016. The range of the MICs was 2 mg/L to 16 mg/L between 2012 and 2015. In 2016, the range was 2 mg/L to 32 mg/L. There were eight isolates with a MIC of 32 mg/L in 2016. Six of these isolates were MDR-GC, the remaining two were only resistant to azithromycin and erythromycin. The above ranges do not include two isolates with a high level of azithromycin resistance (MIC of azithromycin greater than or equal to 256 mg/L), which were identified in 2012 (n=1) and in 2016 (n=1). Both high-level azithromycin resistant isolates were classified as MDR-GC. In 2016, azithromycin resistance was identified in six provinces across Canada with over 90% (n=306/327) identified in Quebec and Ontario (Quebec, 64.5%; Ontario, 28.1%; British Columbia, 2.1%; Alberta, 3.0%; Nova Scotia, 0.9%; and Saskatchewan, 0.3%).

In 2016, 47.1% (n=2,136/4,538) of isolates were resistant to ciprofloxacin; 31.7% (n=1,439/4,538) of the isolates were resistant to erythromycin; 17.4% (n=791/4,538) were resistant to penicillin; and 53.3% (n=2,419/4,538) were resistant to tetracycline. Most of these isolates were resistant to more than one antimicrobial. Spectinomycin resistance was not detected in any isolates tested in 2016.

## Discussion

The proportion of MDR-GC isolates in Canada increased between 2012 and 2016. While the proportion of *N. gonorrhoeae* with decreased susceptibility to cephalosporins has decreased, the proportion of isolates resistant to azithromycin has increased, driving the overall increase in MDR-GC. The XDR-GC are rare in Canada and the proportion identified decreased between 2012 and 2016, due to the decline in isolates with decreased susceptibility to the cephalosporins.

In 2013, Canada’s treatment guidelines for uncomplicated gonococcal infection changed from monotherapy with third-generation cephalosporins to combination therapy with ceftriaxone plus azithromycin (6). Once the combination therapy was introduced, a declining trend of decreased cephalosporin susceptibility was identified. The UK, Australia and the United States (US) have reported similar trends. Combination antimicrobial therapy (ceftriaxone 500 mg IM and azithromycin 1 g orally, in a single dose) was recommended for treatment of uncomplicated gonococcal infections in the UK in 2011 (35). After implementation of the new guidelines, isolates with decreased susceptibility to cefixime declined significantly from 10.8% in 2011 to 5.2% in 2013 (36) and then to 0.6% in 2015 (37). Australia also changed their recommended treatment guidelines (to 500 mg ceftriaxone plus 1 g azithromycin) in 2013 (38). The proportion of isolates with decreased susceptibility to ceftriaxone declined from 4.4% in 2012 (39) to 1.1% in 2017 (40). The recommended therapy for uncomplicated gonococcal infections in the US was updated to ceftriaxone (250 mg IM) combined with azithromycin (1 g orally) in 2012 (41). In the US, decreased susceptibility to cefixime declined from 0.9% in 2012 to 0.3% in 2016 and decreased susceptibility to ceftriaxone remained stable at 0.3% in 2012 and 2016 (42).

While the proportion of decreased susceptibility to cephalosporins has decreased in Canada, the proportion of azithromycin-resistant isolates has increased to 7.2% in 2016 (5), with the majority identified in Quebec and Ontario. Once antimicrobial resistance is established in a region, there is a high risk of these isolates spreading into neighbouring jurisdictions via social networks (43). In 2016, the level of resistance exceeded the 5% level at which the World Health Organization recommends reviewing and modifying national guidelines for treatment of sexually transmitted infections (25). Australia reported similar levels of azithromycin resistance (9.3% in 2017) (44) to Canada; however, the levels in the US (3.6% in 2016) (37) and the UK (4.7% in 2016 [MIC greater or equal to 1 mg/L]) (45) were lower.

The UK and Australia recently reported treatment failures due to high-level XDR-GC with ceftriaxone (MICs=0.5 mg/L) and high-level azithromycin resistance (MIC greater than or equal to 256 mg/L) (24). These strains of XDR-GC threaten the success of the current recommended therapy. With the emerging risk of ceftriaxone resistance and the increasing rate of azithromycin resistance, the *Canadian Guidelines on Sexually Transmitted Infections* has added an alternative combination therapy (gentamicin, 240 mg IM plus azithromycin, 2 g oral) to the list of recommended gonococcal therapies (6).

### Strengths and limitations

The strength of this study is that it is a national laboratory-based surveillance system that can identify changing trends in gonococci antimicrobial resistance patterns over time. The limitations of this study include the representativeness of isolates collected in a passive surveillance system, which may be biased towards cultures isolated from specific populations seeking treatment at clinics that provide culture diagnostics. This could lead to considerable missing data concerning affected populations. The epidemiological data are limited and there is a lack of data pertaining to risk factors and demographics. In Canada, cultures were only available for approximately 22% of reported cases for this study period and the remaining cases were diagnosed using NAAT (5); for these cases, antimicrobial susceptibilities were unknown. In addition, the provinces collect cultures according to their own provincial guidelines and perform antimicrobial susceptibility testing using various susceptibility-testing methods.

### Next steps

To address the lack of surveillance data in the jurisdictions that have data only from NAAT, the NML developed assays that can be used to predict antimicrobial resistance and sequence type directly from NAAT specimens (46–48). While these assays cannot replace culture-based MIC determinations, they can aid in surveillance by predicting antimicrobial susceptibilities to cephalosporin, ciprofloxacin and azithromycin and, together with molecular typing, can provide an understanding of the types of gonorrhea circulating in a community. This work is not routinely performed but is reserved for remote regions where bacterial culturing is not possible.

To address some of the limitations associated with the national passive laboratory surveillance program, PHAC launched the Enhanced Surveillance of Antimicrobial Resistant Gonorrhea in 2013 (49). Laboratory data, such as antimicrobial susceptibility data and sequence typing, are linked to enhanced epidemiological data, which includes demographic and clinical information, risk behaviours, infection site and prescribed treatment information (49). This enhanced laboratory-epidemiological linked surveillance program is currently being conducted in several provinces with plans to expand to other jurisdictions. These data will improve the understanding of antimicrobial-resistant *N. gonorrhoeae* in Canada and provide better evidence to inform the development of treatment guidelines and public health interventions.

## Conclusion

Although rates of MDR-GC increased between 2012 and 2016, XDR-GC in Canada is currently rare. The data presented in this report support efforts to limit the spread of antimicrobial-resistant *N. gonorrhoeae* and prevent the emergence of XDR-GC. In some parts of Canada, azithromycin-resistant GC have exceeded the 5% level at which the World Health Organization recommends reviewing and modifying treatments. Continued surveillance of gonococcal antimicrobial susceptibilities is vital to inform treatment guidelines and mitigate the spread of MDR-GC and XDR-GC.
